# Wavelength- and irradiance-dependent changes in intracellular nitric oxide level

**DOI:** 10.1117/1.JBO.25.8.085001

**Published:** 2020-08-12

**Authors:** Nathaniel J. Pope, Samantha M. Powell, Jeffrey C. Wigle, Michael L. Denton

**Affiliations:** a Oak Ridge Institute of Science and Education, Air Force Research Laboratory, Joint Base San Antonio Fort Sam Houston, Texas, United States; b National Research Council, Air Force Research Laboratory, Joint Base San Antonio Fort Sam Houston, Texas, United States; c Air Force Research Laboratory, Joint Base San Antonio Fort Sam Houston, Texas, United States

**Keywords:** photobiomodulation, nitric oxide, low-level light, low-level laser, retinal pigment epithelium, fluorescence

## Abstract

**Significance:** Photobiomodulation (PBM) refers to the beneficial effects of low-energy light absorption. Although there is a large body of literature describing downstream physiological benefits of PBM, there is a limited understanding of the molecular mechanisms underlying these effects. At present, the most popular hypothesis is that light absorption induces release of nitric oxide (NO) from the active site of cytochrome c oxidase (COX), allowing it to bind $O_{2}$ instead. This is believed to increase mitochondrial respiration, and result in greater overall health of the cell due to increased adenosine triphosphate production.

**Aim:** Although NO itself is a powerful signaling molecule involved in a host of biological responses, less attention has been devoted to NO mechanisms in the context of PBM. The purpose of our work is to investigate wavelength-specific effects on intracellular NO release in living cells.

**Approach:** We have conducted in-depth dosimetry analyses of NO production and function in an *in vitro* retinal model in response to low-energy exposure to one or more wavelengths of laser light.

**Results:** We found statistically significant wavelength-dependent elevations (10% to 30%) in intracellular NO levels following laser exposures at 447, 532, 635, or 808 nm. Sequential or simultaneous exposures to light at two different wavelengths enhanced the NO modulation up to 50% of unexposed controls. Additionally, the immediate increases in cellular NO levels were independent of the function of NO synthase, depended greatly on the substrate source of electrons entering the electron transport chain, and did not result in increased levels of cyclic guanosine monophosphate.

**Conclusions:** Our study concludes the simple model of light-mediated release of NO from COX is unlikely to explain the wide variety of PBM effects reported in the literature. Our multiwavelength method provides a novel tool for studying immediate and early mechanisms of PBM as well as exploring intracellular NO signaling networks.

## 1 Introduction

Photobiomodulation (PBM) is an umbrella term for observed beneficial biological effects of low-energy visible to near-infrared (NIR) light absorption.^1^^,^^2^ PBM has previously been referred to as “laser biostimulation,” “low-level laser therapy,” and “low-level LED therapy,” and typically refers to subthermal red to NIR laser or LED light (though effects in the green and blue wavelength ranges have also been reported).^1^^–^^5^ In contrast to traditional phototherapy, which involves much higher irradiances of UV or full-spectrum light,^6^ or photodynamic therapy, which requires the administration of a light-activated exogenous photosensitizer,^7^ PBM exposures are low energy and usually involve either laser or very narrowband light sources, and are mediated by photon absorption by purely endogenous cell/tissue components.^1^^,^^2^^,^^8^ Reported therapeutic effects of PBM include enhanced tissue healing,^3^^,^^4^^,^^9^^,^^10^ reduction of pain and inflammation,^9^^,^^11^^,^^12^ nerve regeneration,^13^^–^^18^ protection of tissues from poisons of oxidative phosphorylation,^19^^,^^20^ protection from retinal damage due to high-intensity light or hyperoxia,^21^^–^^26^ and amelioration of symptoms of traumatic brain injury.^27^^–^^30^ While there is a rapidly expanding body of published work on these observed benefits, the molecular mechanisms governing the initiation of these effects remain poorly understood. Downstream effects include increased cellular survival and proliferation,^2^^,^^12^ increased adenosine triphosphate (ATP) generation,^2^^,^^8^ and alteration of gene expression.^1^^,^^30^^–^^33^

The putative chromophore responsible for initiating PBM is cytochrome c oxidase (COX); however, this is largely based on inference due to the similarity between the absorption spectrum of COX and the action spectrum of PBM.^1^^,^^2^^,^^24^^,^^34^^,^^35^ COX is also known as complex IV of the mitochondrial electron transport chain.^36^^–^^38^ Since mitochondrial health and high reserve capacity for ATP generation have been suggested to be an indication of overall cellular health and robustness,^39^^–^^41^ it is postulated that light exposure-induced increases in ATP generation may underlie the observed therapeutic and protective effects of PBM exposures.^8^^,^^35^ The proposed mechanism of this increase in ATP generation is that light absorption causes nitric oxide (NO) to come unbound from COX, allowing $O_{2}$ to bind in its place, and thus increase the rate of mitochondrial respiration and ATP synthesis.^1^^,^^2^^,^^24^^,^^42^^–^^44^ A number of studies have detected increases in NO following PBM, and have attributed this increase to either the aforementioned release of NO from COX^41^^,^^44^ or the photorelease of NO from other cellular locations such as nitrosated/nitrosylated proteins.^45^ Additional studies have suggested that COX itself may act as a nitrite reductase to produce NO, and that this activity is enhanced by light absorption.^46^^–^^49^ Although all of these studies have reported increases in NO following light exposure, the source, kinetics, and downstream effects of NO in PBM remain unclear. Due to NO being an extremely potent signaling molecule,^50^^–^^52^ we have conducted an in-depth *in vitro* analysis of NO levels and potential downstream effects following exposure to one or more wavelengths of light.

## 2 Materials and Methods

### 2.1 Cell Culture

All experiments were conducted utilizing cultured human telomerase reverse transcriptase transformed retinal pigment epithelium (hTERT-RPE) cells, which have previously been established as a clinically relevant model system for the human eye and laser eye injury.^53^^,^^54^ hTERT-RPE cells are adherent cells and were cultured in flasks and plates in hTERT-RPE medium: F12/DMEM 50/50 without L-glutamine base medium (Corning, 15-090-CM), with 10% fetal bovine serum (Atlanta Biochem, S11150), and supplemented to final concentrations of 10-mM HEPES buffer (Fisher, BP-299-100), 2-mM L-glutamine (Corning, 25-005-CI), 100-μg/ml (each) penicillin/streptomycin (Corning 30-002-CI), and 50-μg/ml gentamycin (Corning, 30-005-CR). Cells were split at a ratio of 1:20 every 3 to 4 days, after they had reached 80% to 90% confluency. Cells were grown at 37°C in a humidified incubator (Thermo Scientific HERAcell 150i) with 5% ${\mathrm{CO}}_{2}$. To split cells, medium was removed via aspiration with a vacuum flask, and flasks or plates were rinsed with an equal volume of sterile Dulbecco’s phosphate-buffered saline (DPBS, Corning, 21-030-CM). DPBS was also aspirated, and one-tenth of the volume of the culture media of 0.05% Trypsin EDTA (Corning, 25-052-CV) was added to the flask/dish. Cells were incubated with trypsin for 10 min at 37°C, and then hTERT-RPE medium (4× the volume of trypsin) was added to stop the trypsinization process. Cells were dispersed into a single-cell suspension by triturating with a pipette, and then inoculated into a new flask/dish as needed for experiments, or diluted 1:100 in Isoton II diluent (Beckman Coulter) and counted using a Coulter counter (Beckman Coulter, Z1 Coulter Particle Counter).

### 2.2 Light Exposures

**Fig. 1:**
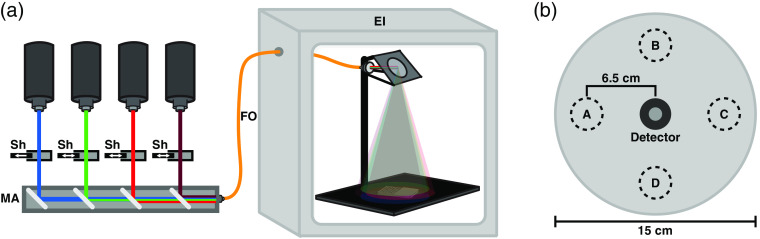
Diagram of laser delivery to sample and method of verifying beam uniformity. (a) Diagram of laser combiner and exposure incubator (EI) used in the PBM experiments. Laser outputs are passed into the mirror assembly (MA) at the bottom-left of the diagram, with shutters (Sh) placed to allow for selection of individual or combinations of wavelengths. Final output to the FO, continues into the EI. Within the incubator, an image of the fiber tip was projected on to a black floorplate to create a 15-cm flat-top laser profile. (b) Diagram depicting method used to verify uniformity of laser irradiance.

Figure 1(a) shows a diagram of the laser delivery setup. Lasers were passed into an assembly of dichroic mirrors placed in series, allowing beams to be coaligned and directed into a single fiber-optic (FO) cable. Laser shutters prior to this assembly allowed for individual adjustment of irradiance for each laser independently. This laser combiner also allowed us to perform serial exposures of multiple wavelengths with ease and accuracy. The FO cable used was a multimode fiber, with 1-mm width and 0.22 numerical aperture. This fiber was fed into a modified cell culture incubator (Thermo Forma Steri-Cult) outfitted with a lens and mirror calibrated to generate a 15-cm-diameter spot size on the exposure area. The input Gaussian beam was homogenized into a flat-top profile at the output face of the fiber because the fiber is multimode. Thus by imaging the output fiber tip onto the sample plane, a uniform flat-top profile was generated. Spot uniformity was confirmed before each experiment by taking laser power measurements across the laser spot in the exposure area, as shown in Fig. 1(b), with a power meter (Newport Power Meter 1918-C) and detector (Newport Silicon Photodiode 918D-SL-OD3R). Variability was never >2%, and detector uncertainty was listed as 1% per manufacturer’s specifications. From these values, the combined error for irradiance measurements was calculated to be 2.24% as per National Institute of Standards and Technology (NIST) recommendations.^55^ Using the $1\text{-}{\mathrm{cm}}^{2}$ window of the power detector, irradiance was adjusted by varying the laser power until the measured irradiance was stable at the desired value. Irradiance was measured again at the end of the exposure to confirm no drift had occurred. Lasers used were as follows—red: High Power Devices 7401 Laser Source, 5-W 635-nm fiber laser; NIR: Laserglow Technologies 5-W 808-nm laser, LRD-0808-PFT-05000-05; green: Laserglow Technologies 2-W 532-nm laser, LRS-0532-PFF-02000-05; and blue: Opto Engine LLC 2-W 447-nm laser, MDL-F-447-2W. Unless otherwise noted, all single exposures were conducted at an irradiance of $800\pm 17.9\;\mu W/{\mathrm{cm}}^{2}$ for 60 min.

**Fig. 2:**
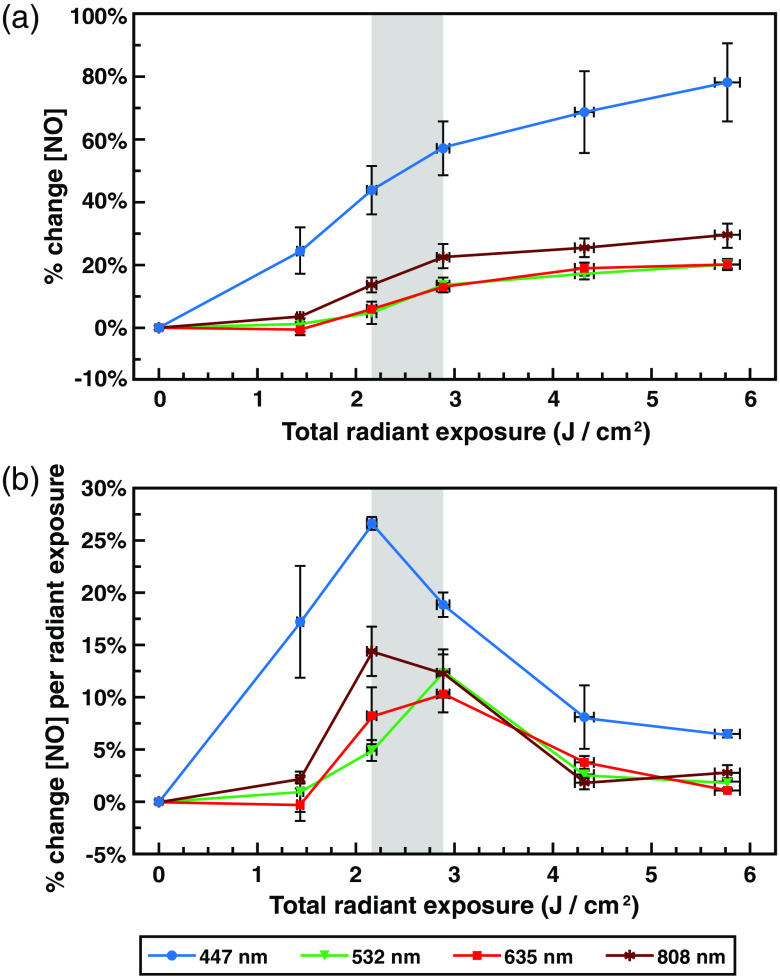
Light exposure increases NO levels in a wavelength-dependent manner, with a biphasic dose–response pattern. (a) Graph of mean percent change (Δf/f DAF-FM fluorescence) ± standard error of NO relative to the total applied radiant exposure following exposures of up to $5.76\pm 0.13\;J/{\mathrm{cm}}^{2}$ of 447-, 532-, 635-, or 808-nm light all at $800\pm 17.9\;\mu W/{\mathrm{cm}}^{2}$. (b) Graph of relative mean percent change (Δf/f DAF-FM fluorescence) of NO per additional $J/{\mathrm{cm}}^{2}$ of radiant energy applied ± standard error, relative to total applied radiant energy (slope of % change NO versus radiant exposure, over total radiant exposure) following exposures of up to $5.76\pm 0.13\;J/{\mathrm{cm}}^{2}$ of 447-, 532-, 635-, or 808-nm light, all at an irradiance of $800\pm 17.9\;\mu W/{\mathrm{cm}}^{2}$. Gray shaded region indicates the (2.16±0.05 to $2.88\pm 0.06\;J/{\mathrm{cm}}^{2}$) maximally responsive exposure range.

The procedure for cell exposures was as follows: a black 96-well plate (Luminunc, Thermo Fisher, 7605) was placed such that the wells to be exposed were in the center of the laser spot, and the unexposed (control) wells were covered with a loosely fitting opaque aluminum foil to block light while not inhibiting gas exchange. In experiments with multiple exposure durations leading to multiple applied radiant exposures (as shown in Fig. 2), this foil was translated by two columns at each time point with two columns remaining covered for the whole duration, allowing for 6 total time-points/radiant exposures and controls. Variations in the amount of time taken to adjust the foil never exceeded 2 sec, so this value was used as the type B uncertainty for exposure duration and was propagated into the uncertainty of the total calculated radiant exposure according to NIST guidelines.^55^

### 2.3 Nitric Oxide Measurements

NO was measured via addition of 4-amino-5-methylamino-2′,7′-difluorofluorescein diacetate (DAF-FM diacetate) (Molecular Probes, D23842), which is a sensitive and specific probe for NO.^56^^,^^57^ DAF-FM diacetate is cell permeable, and once inside the cell is deacetylated by esterases to become DAF-FM. DAF-FM is weakly fluorescent until it reacts with intracellular NO to form a highly fluorescent benzotriazole derivative, increasing in fluorescence ∼160-fold. DAF-FM fluorescence was quantified utilizing a plate reader (TECAN infinite M200 pro) and data were collected using the TECAN iControl 1.10 software, using 486-nm excitation, 515-nm emission, 25 flashes, 70 gain, and 37°C. In all experiments, percent change in NO concentration was reported as the percent increase in DAF-FM fluoresce relative to an equal number of unexposed wells on the same plate (Δf/f DAF-FM fluorescence). As such, control values are incorporated into the presented data and need not be reported separately.

Exposures were conducted in black-bottom 96-well plates with 100,000  cells/well, in 200  μl of DPBS with 5-μM DAF-FM diacetate, and supplemented with 8-mM sodium pyruvate (Fisher, BP356-100) unless otherwise mentioned. The same concentration (8 mM) was also used in succinate (Sigma-Aldrich, S9637-100G) experiments. In the neuronal nitric oxide synthase (nNOS) inhibition experiments, SKF-525A hydrochloride (Calbiochem, 567300) was added to a final concentration of 450  μM. Given this volume and the dimensions of the 96-well plate, cells were distributed within a 5.3-mm-deep cylinder and a diameter of 6.9 mm. This suggests that some cells near the top of the cylinder may have received greater radiant exposures due to attenuation by the cells and medium. However, any potential layering of cells would be expected to cause minimal attenuation due to very low absorption by DPBS and nonpigmented RPE cells in this wavelength range. Cell viability was not directly assayed because damage thresholds for these cells, as previously established by our lab,^53^^,^^54^^,^^58^ were more than 500-fold greater than the irradiances used here. Additionally, any laser-independent cell death that occurred in extended time points would be accounted for in the control wells.

Type A uncertainty (as standard error of the mean) was used for these fluorescence measurements, because random biological and experimental variability was substantially greater than the uncertainty stated by the plate reader manufacturer. Assessment of statistical significance was conducted via unpaired two-tailed Student’s t-test.

### 2.4 Kinetics of NO Release

For NO measurements taken during the laser exposure (as in Sec. 3.3), experiments were conducted differently. Exposures were performed in 12-well plates, which had been coated with 0.5 ml of sterile 3% agar (Sigma, A7049) per well to prevent cellular adhesion to the substratum. Cells were prepared at $1\times 10^{6}\text{ cells}/\mathrm{ml}$ in DPBS with 5-μM DAF-FM diacetate, 8-mM sodium pyruvate, and distributed into 10 of the 12 wells at 0.5 ml per well. The two remaining wells were filled with 0.5-ml DPBS with 5-μM DAF-FM diacetate and 8-mM sodium pyruvate with no cells to determine the baseline fluorescence. One half of the plate was covered with opaque aluminum foil, while the other half was placed centered in the laser spot at an irradiance of 400±8.9, 800±17.9, or $1600\pm 35.8\;\mu W/{\mathrm{cm}}^{2}$. At selected time points during the exposure (15, 30, 60, 90, and 120 min at $400\pm 8.9\;\mu W/{\mathrm{cm}}^{2}$; 5, 15, 30, 45, and 60 min at $800\pm 17.9\;\mu W/{\mathrm{cm}}^{2}$; and 5, 10, 15, 20, and 30 min at $1600\pm 35.8\;\mu W/{\mathrm{cm}}^{2}$), cells were mixed in the well by trituration, and 350-μl aliquots of the cell solution were removed and placed in 1.5-ml snap cap tubes. Aliquots for exposed cells were taken from separate exposed wells, and control aliquots were removed from separate unexposed wells. An equal volume of 100% isopropanol (Sigma, I9516) was added to kill the cells, and the mixture was vortexed and placed on ice until the entire exposure was completed. Tests showed that the addition of isopropanol effectively halted the increase in DAF-FM fluorescence associated with normal live cells, while preserving fluorescence intensity. After all samples were collected, 600  μl of each 700-μl cell/isopropanol mixture was aliquoted into a black 96-well plate, with three replicate wells per condition, at 200  μl per well, and read in the plate reader using the same settings as in Sec. 2.3. These values were averaged, and experiments were repeated in triplicate for each irradiance, providing a total of 9 measurements per time point. Percent increase in DAF-FM fluorescence during laser exposure was calculated relative to fluorescence of corresponding control wells on the same plate (Δf/f DAF-FM fluorescence).

### 2.5 cGMP Measurements

Cyclic guanosine monophosphate (cGMP) concentration was measured using the Cayman cGMP enzyme-linked immunosorbent assay (ELISA) kit (Cayman Chemical Company, 581021). General procedure was as described in the corresponding ELISA protocol included with the assay. To achieve the required sensitivity range of 0.23 to 30  pmole/ml, we calculated that this would require the use of $∼1\times 10^{6}\text{ cells}$. As such, one entire 10-cm dish of cells was used per measurement. Cells were seeded at $0.5\times 10^{6}\text{ cells}/\text{plate}$ 24 h prior to cGMP experiments. At the start of the experiment, medium was removed via aspiration and replaced with DPBS with 8-mM sodium pyruvate to better match the conditions in previous NO measurements. One plate was placed in the laser spot while the other was covered in an opaque foil and placed outside the laser spot. Immediately after the exposure, cells were trypsinized and counted as described previously, and resuspended in 0.1-M HCl (Fisher, S156-1) at $1\times 10^{6}\text{ cells}/\mathrm{ml}$ and incubated for 20 min to produce a cGMP extract. Samples were assayed immediately and compared to a standard curve using the standards provided in the kit. Standards were measured in duplicate, and 4 to 6 replicates of each experimental condition were assayed. Absorbance was measured in the TECAN M200 pro plate reader with the absorbance wavelength set to 412 nm, with 25 flashes (measurements). Experiments were conducted in triplicate. Cells were processed and assayed in the same manner for the cGMP timecourse, with the addition of a 20- and 90-min delay before trypsinization for the corresponding time point.

## 3 Results and Discussion

### 3.1 Light Exposures Induce a Wavelength-Dependent Change in NO

Previous results from this lab demonstrated exposure to $∼2.88\;J/{\mathrm{cm}}^{2}$ of 635-nm red light resulted in a modest increase in NO (10% to 20%) in this *in vitro* RPE system.^59^ Although red (630 to 670 nm) and NIR (780 to 940 nm) wavelengths are the most commonly used in PBM applications,^2^^,^^5^^,^^17^^,^^18^^,^^60^^–^^63^ various studies have provided evidence of PBM effects in the blue and green wavelength ranges.^2^^,^^5^^,^^17^^,^^18^^,^^62^^–^^66^ As such, we conducted assays to determine if any of these wavelengths also had the ability to increase NO levels in this system. We chose 635 and 808 nm as our red and NIR lasers, respectively, as these wavelengths are common in PBM applications. Although green wavelengths are not commonly used in PBM studies, some reports show effects in the green range.^5^^,^^62^^,^^64^ Our choice of 532-nm green was the best available laser that matched this wavelength range with sufficient power. Blue wavelengths are becoming more frequently used in PBM, and while many of these use wavelengths in the 400- to 420-nm range, exposures from 445 to 450 nm are not uncommon,^63^^,^^65^^,^^66^ The 447-nm laser we chose fell roughly in the center of this range and lies near the soret band for COX at 443 nm.^67^ Therefore, we conducted exposures to 635-nm red, 447-nm blue, 532-nm green, and 808-nm NIR lasers (Fig. 2), all at $800\pm 17.9\;\mu W/{\mathrm{cm}}^{2}$ for 30, 45, 60, 90, and 120 min (yielding radiant exposures of 1.44±0.03, 2.16±0.05, 2.88±0.06, 4.32±0.10, and $5.76\pm 0.13\;J/{\mathrm{cm}}^{2}$, respectively).

For all four wavelengths, exposed cells exhibited statistically greater DAF-FM fluorescence compared to control cells at all time points after 30 min, indicating increased levels of free NO. DAF-FM fluorescence rose with applied radiant exposure, until $∼2.88\pm 0.06\;J/{\mathrm{cm}}^{2}$ of total radiant exposure, at which point additional applied radiant exposure had minimal effect. This was particularly evident for 635-nm red, 532-nm green, and 808-nm NIR exposures; however, 447-nm blue exposures appear to continue to rise, though at a reduced rate [Fig. 2(a)].

Once DAF-FM reacts with NO and forms the fluorescent benzotriazole derivative, it remains fluorescent for an extended period of time, and thus represents an integrated, not instantaneous, measurement of total available NO.^56^^,^^57^ As such, we calculated the relative dose–responses by graphing the slope of the DAF-FM fluorescence per additional radiant exposure, relative to total applied radiant exposure, which in essence takes the derivative of the integrated measurement and gives a representation of NO produced between each time point [Fig. 2(b)]. This revealed a biphasic dose–response, with the most effective radiant exposure for all four wavelengths falling at or close to the same $2.88\pm 0.06\;J/{\mathrm{cm}}^{2}$ observed for 635-nm red ($2.88\pm 0.06\;J/{\mathrm{cm}}^{2}$ in the case of 532-nm green, and $2.16\pm 0.05\;J/{\mathrm{cm}}^{2}$ in the case of 447-nm blue and 808-nm NIR). Surprisingly, the increase in NO associated with 447-nm blue light was far greater than any of the other wavelengths measured across the entire range of exposures.

**Fig. 3:**
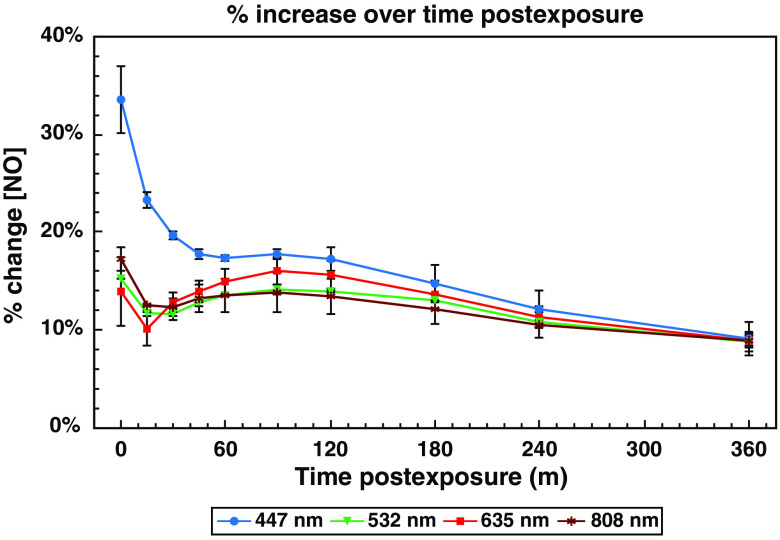
Postexposure kinetics of the light-dependent changes in cellular NO concentration. NO levels remain elevated for several hours following light exposure, peaking immediately following exposure, with a secondary rise ∼90  min later. Graph of mean percent change (Δf/f DAF-FM fluorescence) ± standard error of NO relative to time postexposure to $2.88\pm 0.06\;J/{\mathrm{cm}}^{2}$ of 447-, 532-, 635-, or 808-nm light, all at an irradiance of $800\pm 17.9\;\mu W/{\mathrm{cm}}^{2}$.

With $∼2.88\;J/{\mathrm{cm}}^{2}$ producing a near-maximum effect for all wavelengths, this value was used to compare the effects of light exposure on NO at various time points over the period of 6-h postexposure for each of the different wavelengths (Fig. 3). Immediately following the postexposure, both red and green exposures resulted in ∼15% increase in NO (14.0% and 15.3%, respectively), with NIR resulting in a slightly higher value of 17.2% (although this difference was not statistically significant relative to red or green). The 447-nm blue exposure had a significantly higher effect, with an increase of 33.6% (p<0.001 versus red, blue, and NIR). Raw DAF-FM fluorescence values for exposed cells for all four wavelengths were elevated significantly (p<0.001) relative to controls [Supplemental Fig. S1(a)]. For all wavelengths, the degree of increase in NO levels dropped within 15 min of the end of the exposure, even though they remained elevated (p<0.001) compared to unexposed controls.

At 90-min postexposure, there appeared to be a secondary delayed increase in NO. While the initial postexposure increase in NO varied between wavelengths, the rise at 90-min postexposure for all four wavelengths ended up at ∼15% above baseline. These values were elevated significantly (p<0.001) relative to unexposed controls [Supplemental Fig. S1(b)]. Moreover, exposures to all four wavelengths ended with ∼10% increase in NO at the end of the 6-h observation window. DAF-FM fluorescence for all exposed samples remained significantly elevated (p<0.001) relative to unexposed controls at this time point as well [Supplemental Fig. S1(c)]. Regardless of the magnitude of the early changes in NO, this transient rise in NO appears to induce a secondary effect in all four wavelengths to stabilize the level of NO at ∼10% to 15% over baseline for an extended period. Overall, statistical analysis showed that all exposures significantly elevated (p<0.001) DAF-FM fluorescence at all time points within the 6-h observation window.

It is interesting to find variable effects on NO levels when cells were exposed to light at a variety of wavelengths individually, but we wanted to determine if combinations of wavelengths could modulate the response relative to single wavelengths. If certain wavelengths were absorbed at different molecular chromophores, various combinations of wavelengths might interfere with or enhance the response of another. Additionally, if multiple wavelengths were applied serially, would the order of exposure change the degree of NO elevation? To answer these questions, we utilized a series of dichroic mirrors and shutters (Fig. 1) which allowed us to combine the beams of the various lasers, and have them output through the same optical fiber into our EI. This allowed us to conduct sets of serial and simultaneous exposures with two or more wavelengths.

These questions are not new. Manufacturers are beginning to incorporate multiple wavelengths in their PBM devices.^60^^,^^66^^,^^68^^,^^69^ If combinations of wavelengths were found to have synergistic (or detrimental) effects on NO release, our results would have substantial implications for clinical applications. In line with some of these devices, which combine red and NIR wavelengths, we investigated combinations of 635 and 808 nm. Due to 447-nm blue having the highest individual response, we also wanted to see how it interacted with these longer more commonly used PBM wavelengths. The nearly identical changes in NO when cells were exposed to 635- and 532-nm led us to investigate if the effects of these two wavelengths were additive (contribute interchangeably) or synergistic.

**Fig. 4:**
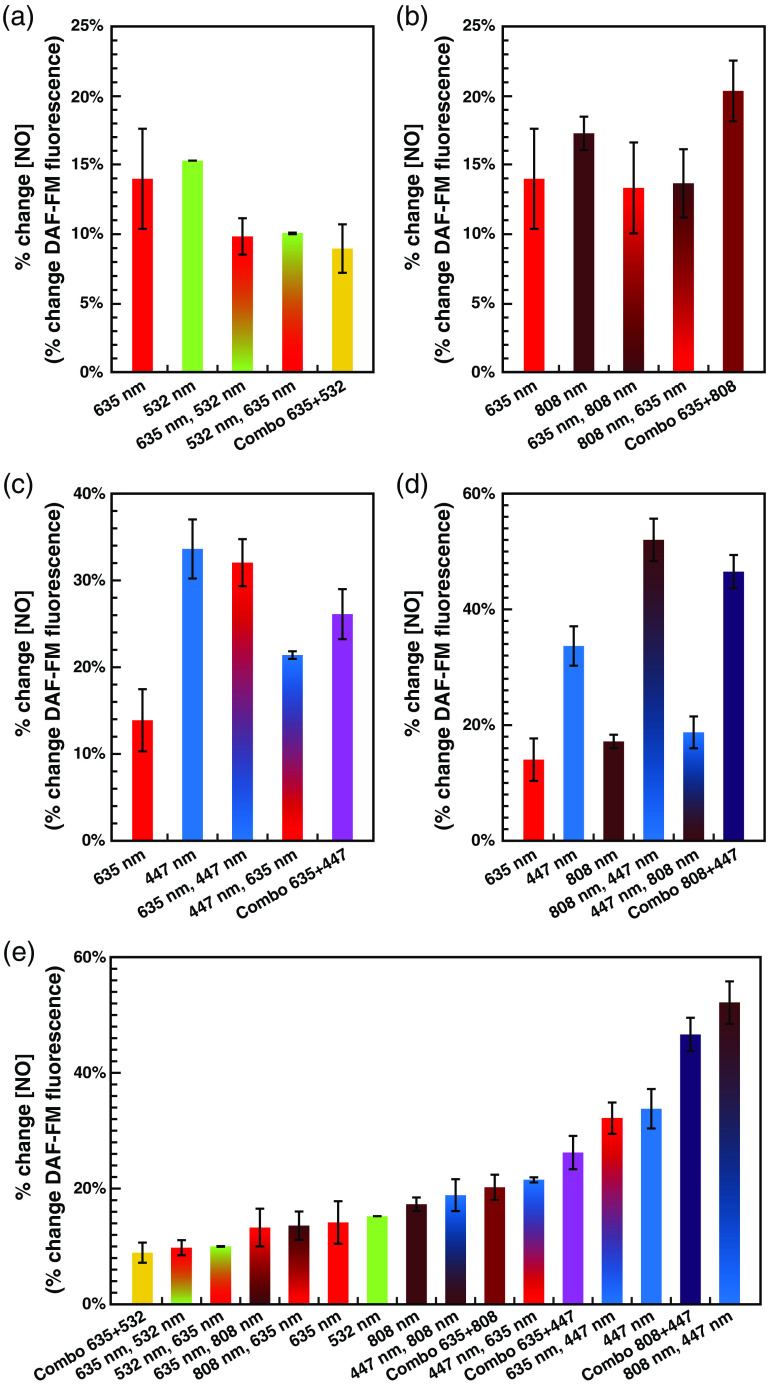
Different combinations of wavelengths (serial or simultaneous) have synergistic and/or interfering effects on the observed light-dependent increases in NO. Individual exposures were of $2.88\pm 0.06\;J/{\mathrm{cm}}^{2}$ at $800\pm 17.9\;\mu W/{\mathrm{cm}}^{2}$ for each wavelength; serial exposures (denoted: first wavelength and second wavelength) were $1.44\pm 0.03\;J/{\mathrm{cm}}^{2}$ ($800\pm 17.9\;\mu W/{\mathrm{cm}}^{2}$ for 30 min) for one wavelength, followed by $1.44\pm 0.03\;J/{\mathrm{cm}}^{2}$ ($800\pm 17.9\;\mu W/{\mathrm{cm}}^{2}$ for 30 min) of the other wavelength (total $2.88\pm 0.06\;J/{\mathrm{cm}}^{2}$); simultaneous exposures (denoted: combo wavelength 1 + wavelength 2) were $1.44\pm 0.03\;J/{\mathrm{cm}}^{2}$ of each wavelength, both at $400\pm 8.9\;\mu W/{\mathrm{cm}}^{2}$, simultaneously for 60 min (total $2.88\pm 0.06\;J/{\mathrm{cm}}^{2}$). Immediate postexposure percent changes in NO level (Δf/f DAF-FM fluorescence) ± standard error for (a) 635-nm red, 532-nm green, and combinations of the two; (b) 635-nm red, 808-nm near-infrared, and combinations of the two; (c) 635-nm red, 447-nm blue, and combinations of the two; (d) 808-nm NIR, 447-nm blue, and combinations of the two (635-nm red is included as a common point of reference); and (e) all of the above wavelengths, in order of percent change.

Examining the immediate postexposure percent increases in NO for these combinations (Fig. 4) reveals several interesting results. First [Fig. 4(a)], although red (n=49) and green (n=52) single exposures were insignificantly different (p=0.73) from one another, the red and green combinations appeared to be less effective [green followed by red (n=72, p<0.001 versus red or green individually), red followed by green (n=71, p=0.067 versus green, p=0.11 versus red), and red and green simultaneously (n=72, p<0.001 versus red or green individually)]. While red followed by NIR (n=40) and NIR followed by red (n=60) serial exposures appear indistinguishable from red alone, the red and NIR simultaneous exposures (n=48) result in a significantly higher increase than that of red (p<0.001) or NIR (n=57, p=0.005) alone [Fig. 4(b)]. Looking at blue and red exposures, serial exposures of red followed by blue (n=48) result in NO levels indistinguishable from blue alone (n=44), while blue followed by red (n=48) was not significantly greater than red alone. The difference between these serial exposures was statistically significant (p<0.001), further demonstrating the order of the exposure is indeed important between these wavelengths. Finally, simultaneous exposures of red and blue (n=96) result in an intermediate value between the two single exposures [Fig. 4(c)].

Blue and NIR combinations [Fig. 4(d)] also result in very different outcomes depending on exposure paradigm. While exposures of blue followed by NIR (n=24) result in NO increases largely identical to NIR alone, and significantly lower than blue alone (p<0.001), NIR followed by blue (n=24) results in an NO increase significantly greater than blue alone (p<0.001). NIR and blue simultaneous exposures (n=48) were similarly higher than blue alone (p<0.001), and while the combination appears slightly lower than the NIR followed by blue serial exposure, this difference is not statistically significant (p=0.22).

In summary, these results imply that the various wavelengths of light potentially modulate NO levels by different mechanisms, and likely different chromophores. Specifically, although red and green individually resulted in the same overall percent increase in NO levels, one was not necessarily capable of substituting for the other. This implies that a full dose of red or green is required for a maximum effect, and that they operate by different mechanisms to drive NO release or production. It is possible that some of these results, particularly the apparent interference of 635 and 532 nm, could be due to different biphasic dose–responses relative to irradiance. If this were the case, two simultaneous exposures at $400\pm 8.9\;\mu W/{\mathrm{cm}}^{2}$ would not necessarily be expected to add up to one exposure of $800\pm 17.9\;\mu W/{\mathrm{cm}}^{2}$. However, given this effect was also observed in the split green–red and red–green exposures, and $1.44\pm 0.03\;J/{\mathrm{cm}}^{2}$ was observed to result in half of the NO release of $2.88\pm 0.06\;J/{\mathrm{cm}}^{2}$ with individual wavelength [Fig. 2(a)], the lack of interchangeability remains surprising.

Even more striking, NIR and blue seem to complement each other when exposed simultaneously, and NIR exposure seems to potentiate the effect of a subsequent blue exposure. Thus, it would seem that to enhance a blue exposure, an NIR wavelength must be present at the beginning of the overall exposure duration. In contrast, blue does not have the same effect on a subsequent NIR exposure, which provides further evidence that different wavelengths are functioning by different mechanisms and chromophores.

An additional byproduct of this analysis is that we have demonstrated that endogenous NO levels can be modulated in finely tuned 2% to 5% increments between 10% and 50% above baseline, utilizing exposures of different combinations of wavelengths [Fig. 4(e)]. Statistically, all of these wavelength combinations produce significantly higher levels of DAF-FM fluoresce relative to their unexposed controls. This will potentially enable novel NO-dose–response experiments to be conducted, notably without the use of NO-donor molecules, which are often light sensitive and thus particularly unsuited for probing mechanisms of PBM.^70^ We intend to utilize this to modulate endogenous levels of NO while looking at downstream signaling mechanisms in the future, although this is beyond the scope of the current paper.

### 3.2 nNOS Activity is Required to Maintain but not Induce Light-Mediated Increases in NO

Next, we interrogated the source of the aforementioned secondary peak in NO levels in the cells at ∼90  min postexposure (as seen in Fig. 3). The putative primary sources of NO in the cell are the nitric oxide synthase (NOS) enzymes. The three isoforms are inducible nitric oxide synthase (iNOS), endothelial nitric oxide synthase (eNOS), and nNOS.^71^ Additionally, some studies have described a mitochondrially localized NOS (mtNOS), which is believed to be an alternatively spliced form of nNOS and is inhibited by nNOS inhibitors.^72^ It is often hypothesized that the immediate source of NO increases in PBM is release of NO from binding sites at COX, in turn allowing for downstream increases in ATP synthesis. However, given the large and long-lasting increases in NO observed (Fig. 2) and the relatively short half-life of NO in the cell,^50^ it is difficult to imagine that this is exclusively due to the release of already extant COX-bound NO. As such, we hypothesized that this initial liberation of NO (or some other PBM-induced secondary messenger) may in turn induce downstream production of NO by NOS enzymes or release of NO from other sources, resulting in long-term increases in detectable NO levels.

**Fig. 5:**
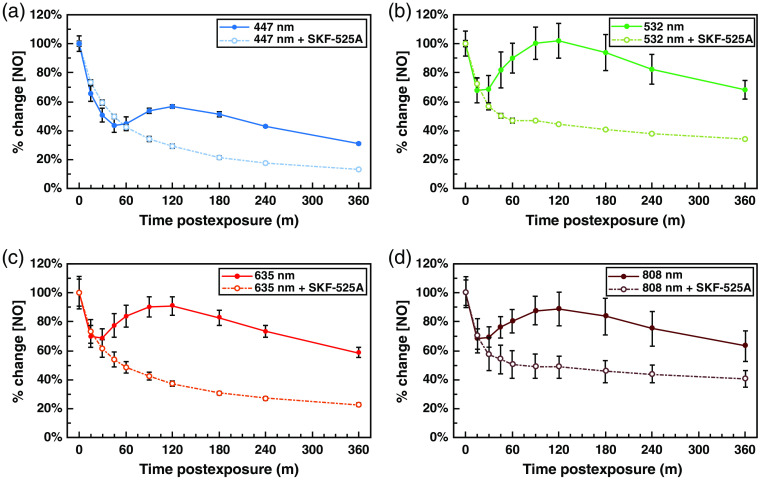
Immediate postexposure increases in NO are nNOS independent, while secondary postexposure increases in NO require nNOS activity. Solid line denotes normal control samples, while dotted line indicates cells treated with 450  μM of nNOS inhibitor SKF-525A. Graph of mean percent changes in NO level (Δf/f DAF-FM fluorescence) ± standard error, relative to time postexposure to $2.88\pm 0.06\;J/{\mathrm{cm}}^{2}$ at $800\pm 17.9\;\mu W/{\mathrm{cm}}^{2}$ of (a) 635-nm red, (b) 808-nm NIR, (c) 532-nm green, and (d) 447-nm blue light.

SKF-525A is a specific inhibitor of nNOS,^71^ and previous work in our lab found that SKF-525A treatment effectively lowers levels of NO in our RPE cell system, while inhibitors of iNOS and eNOS have no significant effect (data not shown). As such, we used SKF-525A treatment to probe if light-dependent increases in NO levels are nNOS dependent. Interestingly, we found that the immediate postexposure NO increases were largely independent of nNOS functionality [Figs. 5(a)–5(d)]. Values were normalized to 100% at the immediate postexposure time point to better compare the relative postexposure kinetics with and without nNOS inhibition. This was necessary because although SKF-525A treatment did not prevent NO levels from rising immediately postexposure, it did lower the baseline levels of NO in the cells. Thus, although light exposure increased NO levels by approximately the same amount with and without SKF-525A treatment based on raw DAF-FM fluorescence, the percentage change was sometimes substantially higher due to the lower starting values (data not shown).

Strikingly, with all wavelengths, the previously observed rise in NO levels seen at 90-min postexposure was completely absent in all cases when nNOS was inhibited. Additionally, NO levels decayed at a much faster rate than in cells without SKF-525A. This unequivocally demonstrates that the observed secondary increase and the long-term elevation of NO following light exposure are dependent on production of NO by nNOS in our cell system. Whether this NOS activity is due to traditionally spliced nNOS, mtNOS, or a combination of the two has yet to be determined.

### 3.3 Midexposure Kinetics and Irradiance Dependence of NO Levels

We have demonstrated that NO levels rise following a complete laser exposure, but we have not investigated the kinetics of NO release during the course of the exposure itself. If NO release from complex IV is a direct photochemical reaction, it should presumably begin immediately following light absorption. However, if changes in free NO are not directly induced by photon absorption and require one or more upstream signaling events, these changes would likely be significantly slower. As such, looking at NO concentrations at various time intervals (kinetics) will provide novel insights into the mechanism underlying the full 60-min increase. Additionally, it is unknown if, or how, irradiance may affect these kinetics. Unlike in Figs. 2–5, to investigate these kinetics, we analyzed NO levels during the course of the laser exposure utilizing the techniques described in Sec. 2.4. Our focus here was on the 635-nm red exposures because, even though 447-nm light induced more NO in our cells, the red light is more representative of traditional PBM exposures. We anticipate pursuing kinetics of NO release from blue light exposure in future experiments.

**Fig. 6:**
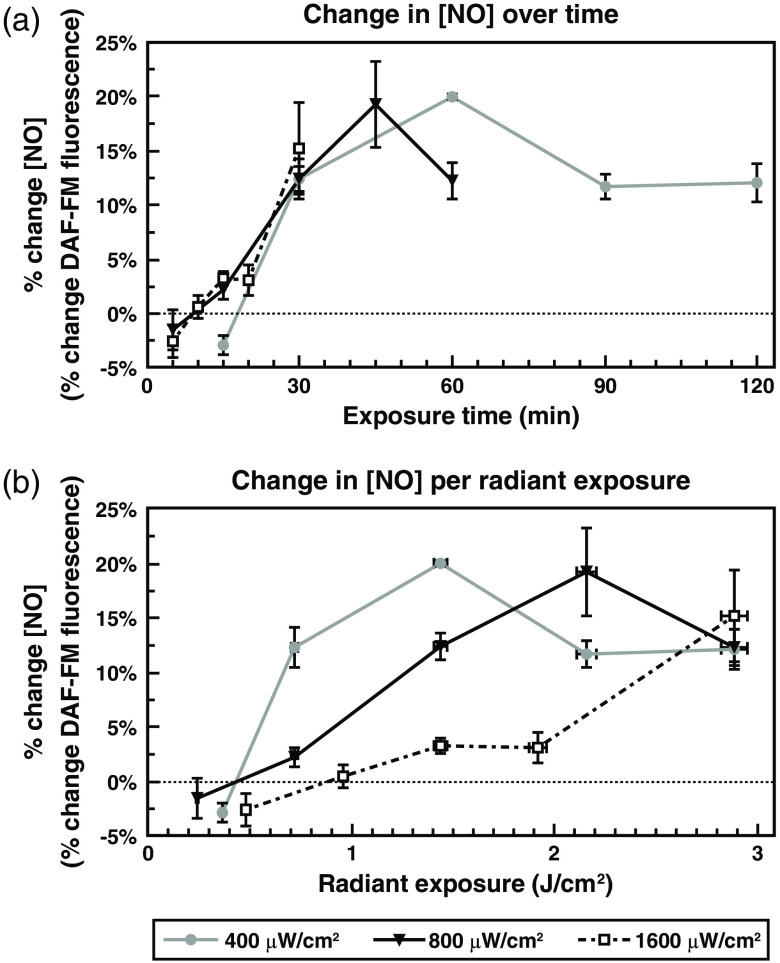
NO levels rise during red light exposure in an irradiance-dependent manner. (a) Graph of mean percent change (Δf/f DAF-FM fluorescence) ± standard error of NO relative to exposure time (min) to 635-nm light at irradiances of 400±8.9, 800±17.9, or $1600\pm 35.8\;\mu W/{\mathrm{cm}}^{2}$ (*p<0.01). (b) Graph of mean percent change (Δf/f DAF-FM fluorescence) of NO relative to radiant exposure in $J/{\mathrm{cm}}^{2}\pm \text{standard error}$ with irradiances of $400\pm 8.9\;\mu W/{\mathrm{cm}}^{2}$ (gray line), $800\pm 17.9\;\mu W/{\mathrm{cm}}^{2}$ (black line), or $1600\pm 35.8\;\mu W/{\mathrm{cm}}^{2}$ (dashed line).

Irradiances of 400±8.9, 800±17.9, or $1600\pm 35.8\;\mu W/{\mathrm{cm}}^{2}$ of 635-nm light were assayed, each with a final radiant exposure of $2.88\pm 0.06\;J/{\mathrm{cm}}^{2}$, necessitating total exposure times of 120, 60, or 30 min, respectively. Percent changes (Δf/f DAF-FM fluorescence) in NO were calculated, and these values were graphed first in relation to exposure time (minutes of exposure), and then in relation to radiant exposure (Fig. 6). This revealed two critical pieces of information. First, as might be expected, when examining the graphs relative to exposure time, the exposures with a higher irradiance achieved their maximal increase in NO levels at an earlier time point [Fig. 6(a)]. Additionally, with all examined irradiances, 50% or more of the total increase occurs between 15 and 30 min of the exposure, and there is no significant change in NO levels before this point. This suggests that there may be an irradiance-independent delay before NO increases are observed. Surprisingly, when examining the graphs relative to radiant exposure [Fig. 6(b)], it becomes clear that lower irradiance exposures result in more efficient per-joule increases in free NO levels in the cells.

### 3.4 Light-Induced and Baseline Levels of NO are Dependent on Mitochondrial Electron Donor Source

**Fig. 7:**
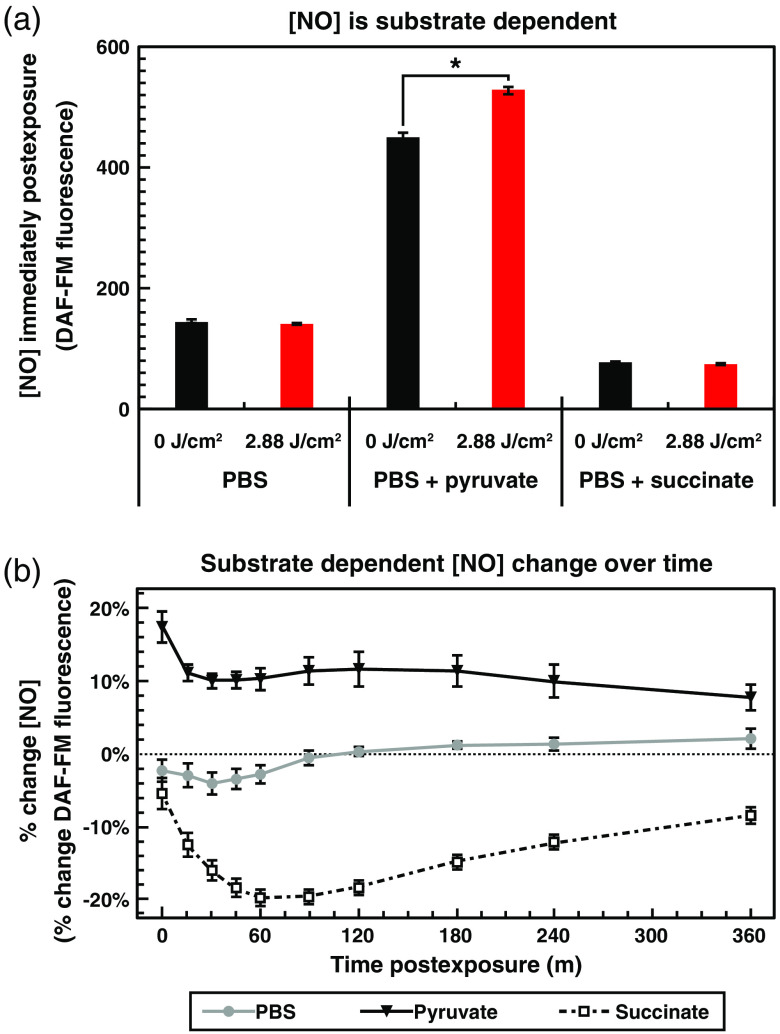
NO levels and PBM-induced changes in NO are dependent on the activity of the mitochondrial electron transport chain. (a) Graph of NO levels (units of DAF-FM fluorescence) with cells in DPBS alone, DPBS with 8-mM sodium pyruvate, or DPBS with 8-mM sodium succinate, each with and without exposure to $2.88\pm 0.06\;J/{\mathrm{cm}}^{2}$ of 635-nm red light at $800\pm 17.9\;\mu W/{\mathrm{cm}}^{2}$. (b) Graph of mean percent change (Δf/f DAF-FM fluorescence) ± standard error of NO relative to time postexposure to $2.88\pm 0.06\;J/{\mathrm{cm}}^{2}$ of 635-nm red light at $800\pm 17.9\;\mu W/{\mathrm{cm}}^{2}$ with cells in DPBS alone (gray line), DPBS with 8-mM sodium pyruvate (black line), or DPBS with 8-mM sodium succinate (dashed line).

Because the immediate postexposure rise in NO levels did not require the activity of nNOS, we were curious as to the source of this NO signal. With COX being the putative chromophore for many PBM effects, a hypothesized source of NO, and a critical component of the mitochondrial electron transport chain, we decided to test if changing the electron source available to the cell would change the NO response. To do so, we compared DAF-FM fluorescence of cells exposed to $2.88\pm 0.06\;J/{\mathrm{cm}}^{2}$ of 635-nm light, in DPBS supplemented with 8-mM pyruvate (as in all of the previous experiments), in DPBS alone, or in DPBS supplemented with 8-mM succinate alone (Fig. 7). Pyruvate and succinate both serve as electron/energy sources for the electron transport chain, but at different points. Pyruvate is the primary metabolic output from glycolysis and is decarboxylated to produce acetyl coenzyme A and nicotinamide adenine dinucleotide (NADH). Acetyl coenzyme A feeds into the tricarboxylic acid cycle, the products of which feed into both complex I (NADH: ubiquinone oxidoreductase) and complex II (succinate dehydrogenase), while NADH feeds into the electron transport chain at complex I.^73^ On the other hand, succinate feeds electrons directly into complex II.^73^ Although we hypothesized that the electron donor source might affect light-mediated changes in NO, we were surprised to see that it also had a dramatic effect on overall levels of NO in unexposed cells.

In the absence of substrate, the cells produced less than a third of the amount of NO at baseline, and did not respond at all to red light exposure [Fig. 7(a)]. Even more surprisingly, the addition of succinate did not restore the production of NO, but rather diminished it further to approximately one-half of the level observed in DPBS alone and to one-sixth of that with the same concentration of pyruvate. Additionally, the effect of red light exposure was dramatically different between the two-electron donor sources. While cells exposed in DPBS showed no statistically significant difference between 635-nm treated and unexposed controls, cells exposed in DPBS with succinate exhibited a decrease in NO levels following 635-nm treatment. This decrease became even more dramatic over time, with a decrease of ∼20% by 1-h postexposure, then stabilizing at ∼10% decrease after 6 h [Fig. 7(b)]. These data indicate that the electron transport chain, and thus the function of the mitochondria, is intimately connected to not only the changes in NO following light exposure but also the baseline level of NO in the cell.

### 3.5 Light-Induced Increases in NO do not Result in Increases in cGMP

While it is generally accepted that NO levels rise after light exposure, the majority of the focus on the role of NO in PBM has been in relation to its putative release from COX. Since we have demonstrated that NO levels can increase substantially over long periods of time, we decided to probe the downstream signaling activity of NO following PBM. The primary signaling target for NO is soluble guanylate cyclase (sGC), which undergoes a conformational change following NO binding, increasing its catalytic activity by more than 2 orders of magnitude.^25^^,^^74^ sGC catalyzes the conversion of guanosine triphosphate to cGMP, a potent second messenger involved in various signaling pathways, including regulation of vascular smooth muscle tone, conductance of various ion channels, and apoptosis.^25^^,^^51^^,^^74^ We theorized that downstream increases in cGMP due to increased levels of NO may underlie some of the observed effects of PBM.

**Fig. 8:**
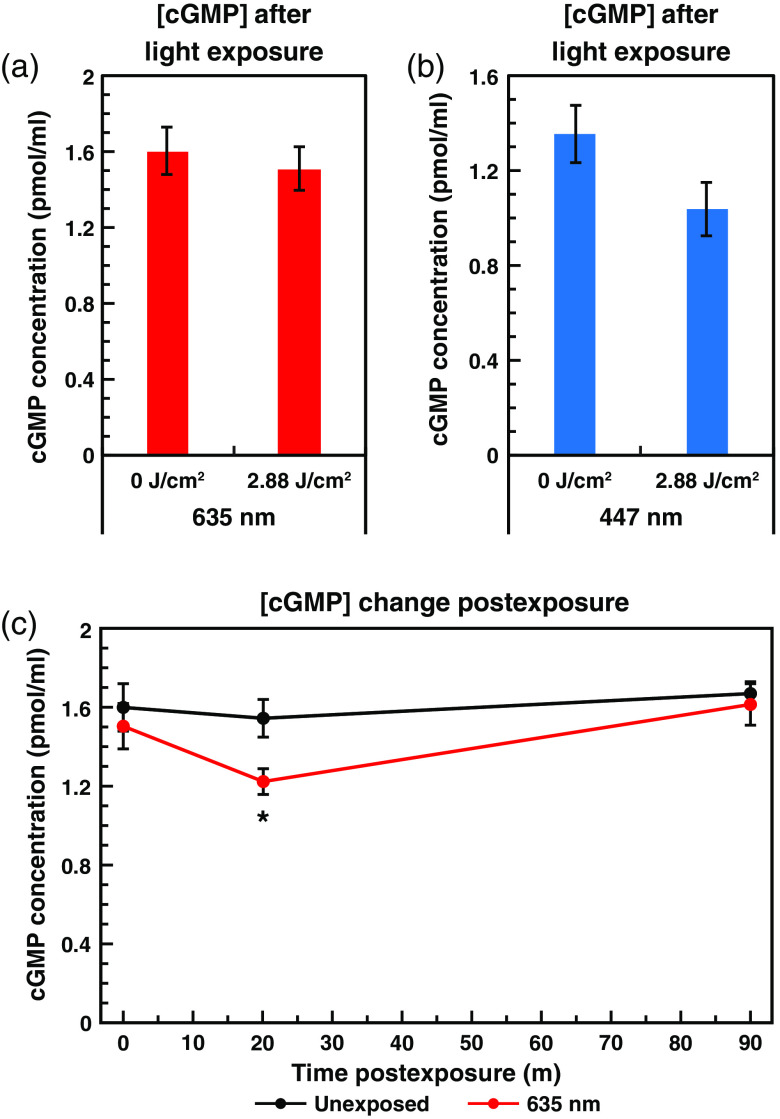
Light-mediated NO increases do not induce cGMP production. Graph of mean cGMP concentration ± standard error as measured by ELISA, with and without exposure to $2.88\pm 0.06\;J/{\mathrm{cm}}^{2}$ at $800\pm 17.9\;\mu W/{\mathrm{cm}}^{2}$ of (a) 635-nm red or (b) 447-nm blue light. (c) Graph of mean cGMP concentration ± standard error, with (red line) and without (black line) $2.88\pm 0.06\;J/{\mathrm{cm}}^{2}$ of 635-nm red light at $800\pm 17.9\;\mu W/{\mathrm{cm}}^{2}$, immediately, 20- and 90-min postexposure. The decrease is statistically significant at 20-min postexposure (p=0.013).

To investigate this, we conducted ELISAs on light exposed and unexposed cells to quantify the levels of cGMP before and after treatment (Fig. 8). Analysis conducted on unexposed control cells versus cells exposed to $2.88\pm 0.06\;J/{\mathrm{cm}}^{2}$ of 635-nm red light [Fig. 8(a)] surprisingly revealed no change in cGMP levels immediately following exposure (p=0.57). We also assayed cells exposed to $2.88\pm 0.06\;J/{\mathrm{cm}}^{2}$ of 447-nm blue light versus unexposed controls [Fig. 8(b)] since the blue light exposure induced the largest increase in NO of the single wavelength exposures (see Sec. 3.1). This condition also exhibited no increase in cGMP levels immediately following exposure. Indeed, there even appeared to be a slight decrease in cGMP, but this decrease was not statistically significant (p=0.065) [Fig. 8(b)].

Since this result was unexpected, we wanted to ensure that there was no delayed increase in cGMP, so we conducted cGMP ELISAs on cells exposed to $2.88\pm 0.06\;J/{\mathrm{cm}}^{2}$ of 635-nm light and unexposed controls harvested 0-, 20-, and 90-min postexposure [Fig. 8(c)]. As with the previous samples, no increase in cGMP was observed at any point postexposure. In fact, at 20-min postexposure, cGMP levels are significantly lower than the unexposed control (p=0.013). The lack of any detectable increase in cGMP despite the observed increases in NO was surprising. However, these observations are not inexplicable, because sGC has been reported to be “desensitized” to NO signals following long-term and/or high magnitude increases in NO. This desensitization is believed to occur via direct modification of cysteine residues by NO, resulting in covalent S-nitrosation.^75^ Additionally, sGC has been reported to be regulated by the cell’s overall redox state, and when sGC is oxidized it can form heme-free apo-sGC, which is unable to be activated by NO.^76^^,^^77^ It is possible that sGC is simply expressed at low abundance, or is largely inactive, in this cell line. Although it would also be instructive to examine other cyclic nucleotide second messengers, including cAMP, which has also been implicated in PBM responses,^78^ it is outside the scope of the current project.

Though cGMP levels did not increase in our system, this does not necessarily imply that NO is not involved in the downstream initiation of beneficial PBM effects. First, the lack of sGC activation and cGMP production observed in our cell system does not necessarily indicate that sGC/cGMP is not activated by PBM in other cell types. Indeed, in their native location, cells of the RPE are in close proximity to both photoreceptors and vascular smooth muscle endothelial cells, which are known to utilize cGMP signaling for regulation of photoreception and vasodilation, respectively. Thus, RPE cells may normally signal predominantly via their neighbors or may have lost their NO signaling capabilities due to the *in vitro* culture setting. Additionally, recent studies have revealed an increasing number of examples of nonclassical (sGC/cGMP-independent) NO signaling,^52^^,^^79^ much of which is through S-nitrosation (sometimes also referred to as S-nitrosylation) occurring predominantly at cysteine residues.^80^^,^^81^ S-nitrosation can occur by direct reaction with NO or via transnitrosation by reaction with a nitrosated intermediate, such as other proteins or glutathione.^82^^,^^83^ Suggestively, S-nitrosation has been implicated in the regulation of a number of proteins previously linked to cell survival and proliferation, including NF-κB,^80^^,^^84^ caspase-3,^80^ p53,^80^ Ras,^80^^,^^85^ several components of the mitogen-activated protein kinase pathway,^80^ all three NOS isoforms,^86^ and complex I of the electron transport chain.^87^^,^^88^ As such, potential PBM-induced changes in S-nitrosation are a topic of interest moving forward, but are outside the scope of the current study.

In summary, we have found that exposure to different wavelengths of light (red, blue, green, and NIR) result in dose-dependent increases in free intracellular NO, with varying magnitudes and efficiencies, depending on the wavelength applied. Furthermore, when multiple wavelengths were combined, either in serial or simultaneous fashion, further differences were revealed. Some wavelengths interfered with or produced a lesser NO response. Specifically, green and red combined exposure liberated significantly less NO than either red or green exposures (both p<0.001) individually. In contrast, other wavelengths worked synergistically and produced a greater NO response than either individually. For example, NIR and red combined exposures liberated significantly more NO than NIR (p=0.005) or red (p<0.001) individually. Likewise, NIR and blue combined exposures liberated significantly more NO than NIR or blue (both p<0.001) individual exposures. We also determined that nNOS activity was not required for the immediate postexposure increases in NO, but is critical for the ability of the light exposure to establish a long-term modest increase in NO levels. We found that red light exposure elevated NO in an irradiance-dependent manner as well, with lower irradiances resulting in more efficient per-joule increases in NO. Figure 7 shows a 70% reduction in the cell’s basal level (no laser exposure) of NO in the absence of pyruvate. When replacing pyruvate with succinate, NO basal levels were reduced by ∼85%. More important, light-mediated increases in NO were not observed in the absence of pyruvate, or when pyruvate was replaced with succinate. As shown in Fig. 8, we found that the increase in NO did not result in a subsequent increase in cGMP.

### 3.6 Conclusions

PBM is a rapidly growing field that is becoming increasingly utilized as a therapeutic treatment and has recently been approved as a first-line medical intervention.^89^ Despite the intense interest, there is still considerable debate over the underlying molecular mechanisms. For over 20 years, COX has been theorized to be the primary chromophore for PBM effects, whereby subsequent NO eviction is the cited mechanism by which light induces changes in mitochondrial function.^1^^,^^8^^,^^35^ Recently, however, that hypothesis has been increasingly challenged,^90^^,^^91^ and a variety of other potential mechanisms and chromophores have been suggested.^2^^,^^92^^–^^95^

This study provides several insights regarding light-mediated release of NO and how it may relate to PBM mechanisms. Our finding that multiple wavelengths of light caused release of intracellularly stored NO provides further support that other mechanisms are in play. This is particularly true since 447- and 532-nm wavelengths are rarely used for PBM. The existence of combinations of wavelengths that interfere (635 and 532 nm) or synergize (808 and 447 nm) with one another suggests that additional chromophores or sources of NO are likely. Our surprising finding that light-mediated (635 nm) NO release required pyruvate suggests that the metabolic state of the mitochondria may play a key role in how our cells respond to photostimulation. Overall, the results of this study support the conclusion that a simple model of light-mediated release of NO from COX is unlikely to explain the wide variety of PBM effects reported in the literature.

Considering the ongoing debate regarding the molecular mechanisms underlying PBM, continued study of NO and its downstream effects are warranted. Using our demonstrated ability to fine-tune the magnitude of NO release, we are conducting dose–response experiments to determine if the concentration of NO correlates to various established downstream PBM outcomes, such as ATP production, mitochondrial membrane potential, or cell division. Having combinations of wavelengths that induce similar or altered levels of NO release, we can begin to uncouple whether or not these downstream indicators of PBM are due to NO, the light, or both. Finally, we are currently pursuing spectroscopic methods to identify whether or not low levels of red/NIR light dislodges NO bound to COX.
