# Supplementary material for: Wavelength- and irradiance-dependent changes in intracellular nitric oxide level
Source: J Biomed Opt. 2020 Aug 12;25(8):085001. doi: 10.1117/1.JBO.25.8.085001 (PMC7423318; doi:10.1117/1.JBO.25.8.085001)
Supplement: Supplementary file 1 [file JBO_025_085001_SD001.pdf]

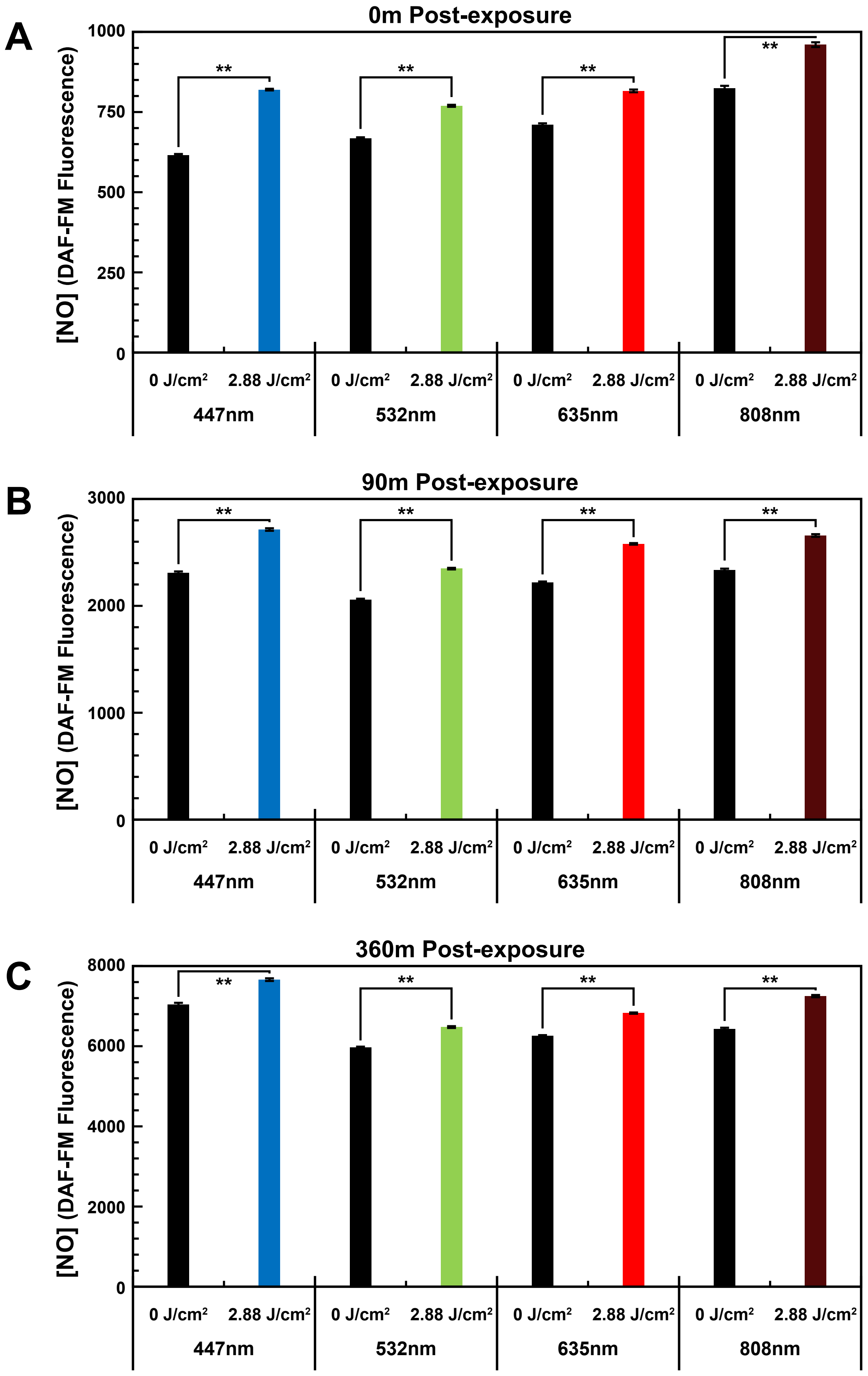

Fig S1. Raw DAF-FM fluorescence values following light exposure. (A) Graph of DAF-FM fluorescence ± standard error immediately following exposure to 2.88 ± 0.06 J/cm2 of 447 nm (n=44), 532 nm (n=52), 635 nm (n=48), or 808 nm (n=56) light (all at an irradiance of 800 ± 17.9 µW/cm2) and corresponding unexposed controls. (B) DAF-FM fluorescence remains elevated at 90 min and (C) 360 min post-exposure. ** $p<0.001$.
